# Cylindrical Free-Standing Mode Triboelectric Generator for Suspension System in Vehicle

**DOI:** 10.3390/mi10010017

**Published:** 2018-12-29

**Authors:** Minki Kang, Tae Yun Kim, Wanchul Seung, Jae-Hee Han, Sang-Woo Kim

**Affiliations:** 1 School of Advanced Materials Science and Engineering, Sungkyunkwan University (SKKU), Suwon 16419, Korea; kang0824@skku.edu (M.K.); herotyun@skku.edu (T.Y.K.); s2000@skku.edu (W.S.); 2 Department of Energy IT, Gachon University, Seongnam 13120, Korea; jhhan388@gachon.ac.kr

**Keywords:** triboelectric generator, shock absorber, suspension system, advanced driver assistance technology, IoT technology, frictional force

## Abstract

The triboelectric generator (TEG) is a strong candidate for low-power sensors utilized in the Internet of Things (IoT) technology. Within IoT technologies, advanced driver assistance system (ADAS) technology is included within autonomous driving technology. Development of an energy source for sensors necessary for operation becomes an important issue, since a lot of sensors are embedded in vehicles and require more electrical energy. Although saving energy and enhancing energy efficiency is one of the most important issues, the application approach to harvesting wasted energy without compromising the reliability of existing mechanical systems is still in very early stages. Here, we report of a new type of TEG, a suspension-type free-standing mode TEG (STEG) inspired from a shock absorber in a suspension system. We discovered that the optimum width of electrode output voltage was 131.9 V and current was 0.060 µA/cm^2^ in root mean square (RMS) value while the optimized output power was 4.90 μW/cm^2^ at 66 MΩ. In addition, output power was found to be proportional to frictional force due to the contact area between two frictional surfaces. It was found that the STEG was made of perfluoroalkoxy film and showed good mechanical durability with no degradation of output performance after sliding 11,000 times. In addition, we successfully demonstrated charging a capacitor of 330 μF in 6 min.

## 1. Introduction

Internet of Things (IoT) technology is becoming more influential within our lives recently. This technology, which is based on wireless sensor network systems, has comprehensive applications, such as health monitoring, smart factories, autonomous driving, etc. Among them, autonomous driving is one of the most promising technologies. As an intermediate stage, a number of automotive companies are trying to develop advanced driver assistance system (ADAS) technology to realize autonomous driving vehicles. As ADAS technology is based on sensor network systems, continuous power supply for stable and long-term operation of sensors is very important. Although individual sensors for sensor networks consume relatively small amounts of energy, ranging from μW to mW, large amounts of energy would be required for the sustainable operation of a huge number of sensors assembled in an IoT system [1,2]. However, current automotive energy harvesting technologies based on traditional electro-magnetic induction are not efficient for powering sensors because of their heavy weight when compared to their output performance, and thus the absence of an efficient energy source for the sensor network system, not only leads to a reduction in energy efficiency of the vehicle, but also disturbs the practical application of ADAS technology in a vehicle [3,4].

Triboelectric generators (TEGs) that convert mechanical energy to electrical energy utilize a combination of triboelectrification and electrostatic induction, which is one of the most promising candidates as an energy source of low-power sensors because of its high output performance, light weight, and low cost [5,6,7,8]. Based on these attributes, there have been numerous attempts to harvest mechanical energy which is abandoned in vehicle such as energy from wind, vibration, rotation and etc through applying TEGs [9,10,11]. However, TEGs that rely entirely on mechanical friction may deteriorate the energy efficiency of vehicles by causing more energy consumption, which is required for driving. For example, a rotating free-standing mode TEG applied to the wheel makes a vehicle consume more energy due to the continuous mechanical friction-induced driving resistance. Alternatively, when a TEG harvesting wind energy blows it into a vehicle, it results in high air resistance that disturbs driving, and thus consumes more energy [9,10,11]. Therefore, it is an important issue to design appropriate structures and harvest inevitably-wasted energy without reducing energy conversion efficiency and the reliability of the vehicles.

Here, we suggest that suspension-type free-standing mode TEG (STEG) can be applicable to reciprocal movement of a shock absorber in the suspension system of vehicles. The frictional force of STEG can perfectively replace the damping force of a conventional shock absorber and does not cause additional energy consumption. STEG has a novel, cylindrical, free-standing mode TEG structure that consists of an aluminum inner cylinder and outer cylinder. The triboelectric charges generated by the friction between a perfluoroalkoxy (PFA) film and grating-structured outer cylinder induce electrostatic induction to the alternating two electrodes on the inner cylinder and generate displacement current. We validated output performance of the STEG, which has a 1:8 scaled size of a real shock absorber in the vehicles, as a function of width of the electrode and speed of the reciprocal movement. In previous reports, many factors that affect the generating performance of TEGs have been investigated, such as charge density, permittivity, thickness of film, and surface area. However, the frictional force between two different materials in sliding mode STEGs have not yet been considered because of the complexity of phenomenon and difficulties in control. Frictional force is proportional to normal force, and the effective contact area increases as the normal force becomes stronger. We obtained the highest output performance of STEG at 4.90 μW/cm^2^ by controlling the frictional force with different radii of the inner cylinder.

## 2. Materials and Methods 

### 2.1. The Fabrication Process of the STEG

The STEG is comprised of an inner cylinder and an outer cylinder made of aluminum with a 1:8 scale sized shock absorber in vehicles. The diameter of the inner cylinder was 23 mm and was surrounded by copper electrodes deposited on a flexible printed circuit board (PCB) (substrate is polyimide, 12.5 μm thick). The copper electrodes (12.5 μm thick) had an interdigitated structure with a 1-mm gap between adjacent electrodes in order to prevent short circuits, and were deposited on the same area of 70 × 130 mm^2^ to investigate the optimum design. Polyethylene (PE) foam tape (1 mm thick) was inserted between the inner cylinder and the surrounding copper electrode-deposited PCB to increase the outer diameter of the inner cylinder and control the frictional force between the inner cylinder and the outer cylinder. Lastly, PFA film, which is a frictional layer with a thickness of 25 μm, was attached to the copper electrode using commercial double-sided tape. The inner diameter of the outer cylinder is 26 mm. The inner surface of outer cylinder which is facing the PFA film on the inner cylinder has a grating structure with the same width as the copper electrode. Both the inner and outer cylinders have lengths of 220 mm.

### 2.2. Characterization of STEG

Output performance of STEG was measured while the inner surface of the outer cylinder slides on the PFA film surface by applying a cyclic vertical force using pushing tester (ZPS-100, JUNIL TECH Co., Ltd., Seoul, Korea). The inner cylinder was fixed on the bottom stage and the outer cylinder was attached to the moving part, which periodically moved with a constant speed of 62.5, 140, and 200 mm/s, respectively, and the period was 0.4 s for all cases. During movement, the frictional force controlled by PE foam tape was measured using a force sensor installed in the pushing tester. The output voltage between two interdigitated electrodes was measured using an oscilloscope (DPO 3052, Tektronix, Beaverton, OR, USA) with an input impedance of 40 MΩ, and the output current was measured using a current amplifier (DLPCA-200, FEMTO, Berlin, Germany) connected to the oscilloscope under short circuit conditions. After 11,000 periodic movements with a frictional force of 0.6 kgf, the surface morphology of PFA film was examined using scanning electron microscopy (JSM-6701F, FE-SEM, Jeol Ltd., Mitaka, Tokyo, Japan) to check mechanical durability.

## 3. Results

### 3.1. Geometrical Design of the STEG and Electrical Performance

The STEG was designed based on the structure of a free-standing mode TEG, in which the copper electrodes on the inner cylinder and outer cylinder corresponded to alternative electrodes and moving objects, respectively. For the investigation and fabrication of a STEG that is compatible with the current suspension systems in vehicles, the materials were selected to satisfy, not only the triboelectric series, which indicates how much the material has tendency to have a positive or negative surface charge, but also to maintain the reliability of the suspension system. 

**Figure 1:**
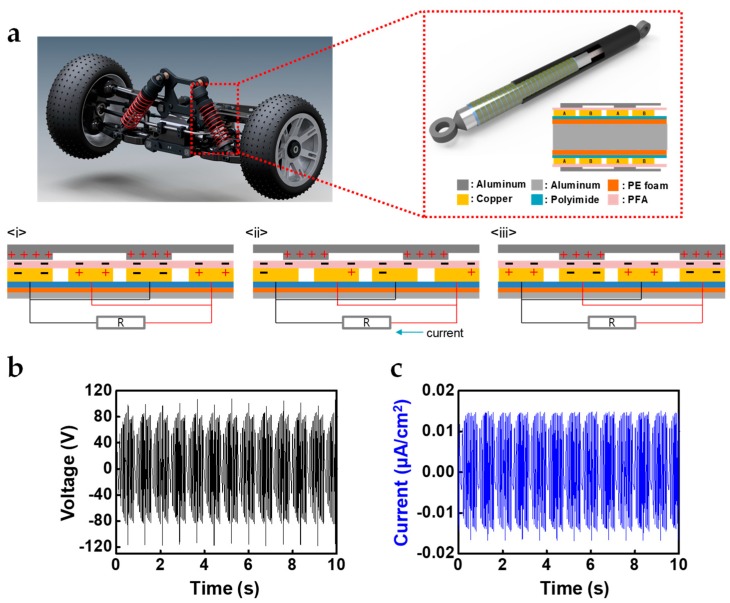
(**a**) Schematic structure and triboelectric regenerative suspension application and the working mechanism of a suspension-type free-standing mode triboelectric generator (STEG); (**b**) the output voltage and (**c**) short-circuit current according to the reciprocating movement of the outer cylinder with a displacement of 50 mm and speed of 100 mm/s.

The schematic structure and triboelectric energy regenerative suspension application is featured in Figure 1a. Each cylinder was made of aluminum, and flexible PCB comprised of polyimide (PI) substrate and alternating copper electrodes was covered by a perfluoroalkoxy film (PFA) on the inner cylinder. Optical images, material, and structure of STEG are shown in Figure S1. The outer cylinder had a grating structure on the inner surface and a slide along the PFA film, generating triboelectric charges. The size of the cylinder was 1:8 scaled size compared to commercial shock absorbers in the suspension systems of vehicles. 

The working mechanism of STEG is based on that of a free-standing mode TEG and is illustrated in Figure 1a(i)–(iii) [12]. The sliding motion of two cylinders leads to triboelectrification due to the friction between the PFA film and the outer cylinder. Subsequently, the PFA film and aluminum outer cylinder have opposite negative and positive charges on their surfaces, respectively, due to their different triboelectric polarities and the amount of transferred opposite charges was saturated. The surface charge density of the outer cylinder was twice the surface charge density of the PFA film according to the law of charge conservation, because the contact area of the outer cylinder is two times smaller than that of PFA film. Under the short-circuit conditions, when the outer cylinder was placed on electrode A, the electrical potential difference between the two electrodes was positively maximized (i). When the outer cylinder slid and was placed between electrode A and B, the current flowed from electrode A to electrode B to compensate for the electrostatic induction and made the electrical potential of electrode A equal to that of electrode B (ii). Subsequently, when the outer cylinder reaches electrode B, the electrical potential difference between the two electrodes would be negatively maximized (iii). These stages would be repeated by reciprocating motion of cylinders. PFA film is known to have, not only the most negativity in triboelectric series (so that the largest amount of charges is generated by triboelectrification), but also abrasion resistance and mechanical durability [13,14,15]. The novel triboelectric and mechanical properties of PFA allow for STEG to have a high output performance with a very long lifetime [16,17]. During reciprocating movement of the outer cylinder with a displacement of 50 mm and speed of 100 mm/s, STEG generated a maximum voltage and current of 100 V and 0.014 μA/cm^2^, respectively, when the damping force was 0.6 kgf and the width of electrode A and B were 3 mm (Figure 1b,c). Since STEG had a cylindrical shape and a frictional force against mechanical movement, it could take a role of a damper for the applied force as well as energy harvester. This point is very important because it means that STEG can harvest wasted energy without unnecessary energy consumption.

**Figure 2:**
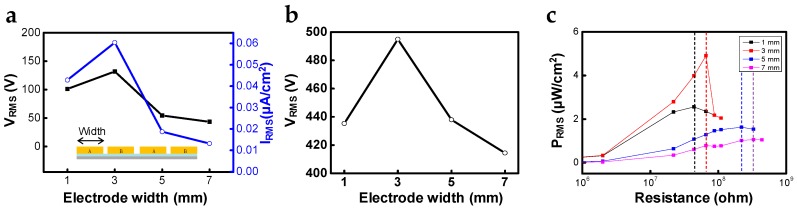
Output performance evaluation and simulation versus electrode width: (**a**) output voltage and short-circuit current; (**b**) COMSOL simulation result (open-circuit voltage) with a resistance of 100 GΩ for open-circuit condition; (**c**) output power in root mean square (RMS) value and different optimum resistance according to changes in the electrode width.

In order to optimize the output performance of STEG, we investigated the dependence of the output performance on geometrical parameters, such as electrode width. We fabricated several STEGs with different widths of copper electrodes (from 1 mm to 7 mm), but the same gap distance of 1 mm between adjacent electrodes. The output voltage and current were measured by sliding the outer cylinder of each STEG at 200 mm/s, with a damping force of 0.6 kgf and displacement of 50 mm, as shown in Figure 2a. Output voltage and current increased as the electrode width increases from 1 mm to 3 mm, showing a maximum value of 131.9 V and 0.060 μA/cm^2^ in root mean square (RMS) value, respectively. However, output voltage and current decreased as the electrode width increased over 3 mm.

The dependence of the output performance on the electrode width in the experimental results was verified using numerical simulations with the same geometric modeling as the experiment. Each electrode, of which the electrode widths were 1, 3, 5, and 7 mm was periodically arrayed and terminated as alternating electrode A and B. The gap distance between neighboring electrodes was 1 mm, and between the moving object and bottom electrode was 25 μm. Experimentally, there was PFA film between the top and bottom electrodes, and triboelectric charges existed on the top surface of the PFA film; however, for simplification, PFA film was not considered and the triboelectric charges (25 μC/m^2^) existed on the bottom surface of the moving object. For calculating the electric voltage between electrodes A and B, while the top object was moving at 50 mm/s, the electrical circuit module was coupled. The resistance of the electrical circuit module was set to 100 GΩ for open-circuit conditions. RMS voltage values of devices with different electrode widths are compared in Figure 2b. Corresponding to the experimental results, simulation results showed the highest voltage value when the electrode width was 3 mm. With a fixed gap distance between the electrodes (1 mm), the electrodes were more separated from neighboring electrodes as the width decreased. In the case of an electric field from the charged plane, the electric voltage was proportional to distance; thus, electric voltage increased as electrode width decreased. However, the electrode has finite area, so the edge effect should be considered. As the electrode width decreased, the edge effect became larger compared with the electric field from the charged plane; thus, the electric field became smaller [18,19]. Considering these two effects, 3 mm was the optimum width of the electrode for the highest output performance. The experimental raw data and simulation results related to structure-dependent electric field are illustrated in Figure S2.

Theoretical study on the electric fields in the different geometries offer us an understanding of the reason why STEG could have high output characteristics. To compare the intensity of electric fields and the potential difference versus geometry of the free-standing mode TEG, a COMSOL simulation was conducted on the planar and cylindrical models with the same area and charge density (Figure S3). When the planar model has the geometry infinity plane, the edge effect could be ignored. However, the edge effect is not negligible as there is a non-continuous adjacent charge at the edge of the plane [20]. This characteristic of the planar model induced decreases in the electric field and potential difference (Figure S3a). On the other hand, in the case of a cylindrical model, the edge effect was negligible because the electric field was distributed radially so that they had higher potential differences with the same area and charge density (Figure S3b). A cross-sectional profile of the electrical potential along the surface is compared in Figure S3c. The cylindrical charged surface exhibits larger potential than the planar charged surface and the difference is 100 V or more.

In addition, we determined that each STEG with electrode widths of 1, 3, 5, and 7 mm had different optimum resistances of 44 MΩ, 66 MΩ, 220 MΩ, and 330 MΩ, respectively, as depicted in Figure 2c. As a result, the STEG with an electrode width of 3 mm could generate 4.90 μW/cm^2^ at 66 MΩ. Electrical impedance of the capacitor was inversely proportional to the frequency and capacitance [12,21]. As the electrode width increased, it takes more time for a contacting part of the outer cylinder to move from electrode A to B; as such, the frequency decreased at the same speed when the effective gap between neighboring electrodes increased. Therefore, the optimum electric impedance of STEG increased as a function of electrode width.

### 3.2. Evaluation of Output Performance depending on Mechanical Input Parameters

**Figure 3:**
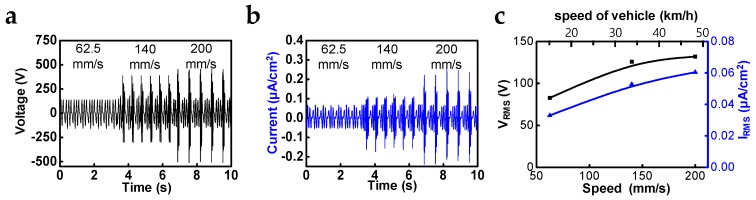
Output performance evaluation versus the different speeds of outer cylinder: (**a**) output voltage and (**b**) short-circuit current with speeds of 62.5, 140, 200 mm/s, respectively; (**c**) output voltage and short-circuit current in RMS value versus the speed of outer cylinder and the speed of a vehicle(expected from the speed of outer cylinder).

**Figure 4:**
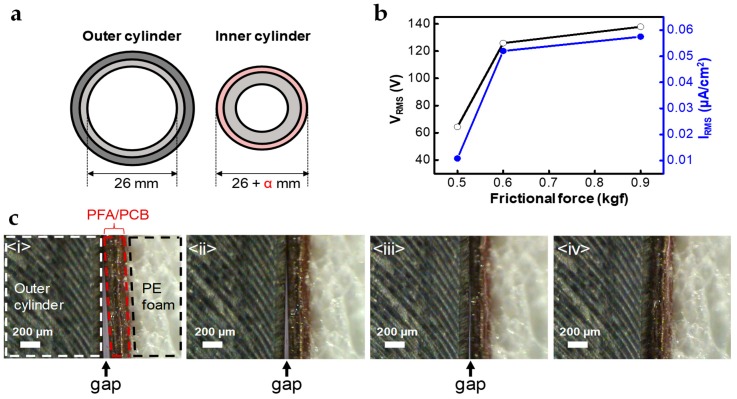
Frictional force as a parameter for output performance of STEG: (**a**) schematic experimental setup to control frictional force through radius control of inner cylinder; (**b**) short-circuit current in RMS value according to increase of frictional force; (**c**) observation by optical microscopy of shape-adapted deformation of PFA film with different α, (i) 0.08 mm, (ii) 0.09 mm, (iii) 0.10 mm, (iv) 0.11 mm, respectively. For α of 0.11 mm, there is no gap.

Faster speeds of reciprocating movements induced higher output performances because of the short-period of electrostatic induction. The output measurements according to changes in the speed were conducted for STEG, which has a damping force of 0.6 kgf when the displacement is 50 mm and the electrode width is 3 mm (Figure 3a,b). As a result, we found that the maximum short-circuit current and output voltage of 0.060 μA/cm^2^ and 131.9 V, respectively, in RMS values could be acquired under a speed of 200 mm/s (Figure 3c). According to reference, which dealt with energy regenerative suspension, the RMS speed of the shock absorber and the speed of a vehicle have a proportional relationship. Through the conversion operation from the speed of the shock absorber to that of a vehicle, the expected short-circuit current in average city driving (32.2 km/h) is about 0.053 µA/cm^2^ in RMS value. Although the frictional force is one of the notable factors that has a great influence on the output performance of TEGs, experimental and theoretical studies have not been reported in detail. There have been several reports dealing with changes of output performance in contact mode TEGs depending on increase of applied pushing force [8,22], but the effect of frictional force on sliding mode TEGs has not been investigated because the structural design needed to control and maintain normal force is complex and too strong a frictional force can cause mechanical damage or a reduction in output power due to the disturbance of the movement. However, the cylindrical structure of STEG enables easy control of the frictional force by attaching a polymer elastomer, such as PE foam tape, between the outer surface of the inner cylinder and the flexible PCB layer, as described in Figure 4a. Without PE foam tape, the diameters of the inner and outer cylinders are matched (inner cylinder diameter is slightly smaller than that of outer cylinder), so additional PE foam tape increases the normal and frictional forces according to its thickness. The α is supposed to be the thickness of additional PE foam, and the frictional force increased to 0.5, 0.6, and 0.9 kgf as α was adjusted to 0.08, 0.18, and 0.38 mm, respectively. The output voltage, short-circuit current and frictional force were measured while sliding outer cylinder at a speed of 140 mm/s with a displacement of 50 mm. The output voltage and short-circuit current (RMS value) as a function of frictional force is shown in Figure 4b, and the maximum output voltage and short-circuit current were 137.9 V and 0.057 µA/cm^2^, respectively (RMS), with saturating behavior. Experimental raw data related to this issue are illustrated in Figure S4.

Due to the elasticity of PE foam tape, it pushes upper flexible PCB and PFA layers to the outer cylinder, which increases the contact area between them, as well as the frictional force. Figure 4c shows a cross-sectional image of the interface between the outer cylinder and the PE foam tape covered by PFA and flexible PCB. The contact area became larger as the thickness of the PE foam tape increased. Triboelectric charges are generated by contact and sliding between the two surfaces. In other words, a larger contact area can generate more triboelectric charges and then output power can be enhanced. However, because the increase in effective area by the applied normal force has certain limitation, the output power vs. frictional force curve has a saturating behavior (Figure 4b). Moreover, too large normal force and frictional force can induce the tearing of the PFA film or disrupt the reliable operation of the suspension. Therefore, we have to choose an optimum frictional force to achieve both high performance and sustainable operation. If the STEG is applied to the suspension system of the vehicle, we can expect much higher output performance because of the damping force of tons’ scale and larger size of shock absorber. Considering these expectations, we can observe the feasibility of STEG as a practical energy source for sensors in ADAS technology. Further study should be conducted considering the decay of output performances under high temperature conditions and when facing abrasion problems. 

**Figure 5:**
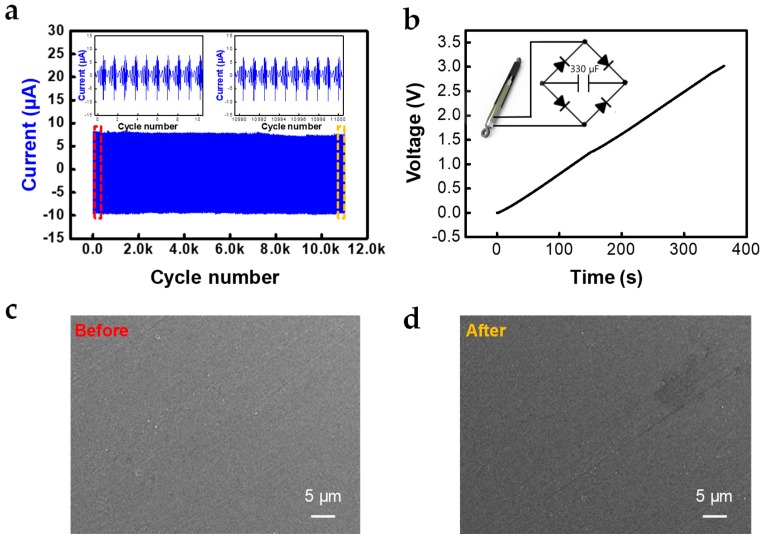
Stability of output performance and charging performance: (**a**) durability test for 11,000 cycles with damping force of 0.6 kgf, speed of 140 mm/s and displacement of 50 mm (inset: short-circuit current signals at the beginning and the end of durability test, respectively; red box is the beginning part and orange box is the end part); (**b**) charging curve for 330 μF using a rectifying bridge, with a frictional force of 0.6 kgf and speed of 200 mm/s; (**c**,**d**) SEM images of surface morphology of PFA film before and after the durability test, respectively.

Based on the results of evaluations, a durability test was conducted and we succeeded in charging the capacitors (Figure 5a,b). STEG maintained a stable output performance for 11,000 cycles, even with a damping force of 0.6 kgf, speed of 140 mm/s and a displacement of 50 mm. Inset figures are the short-circuit current peaks at the beginning (red box) and end of the durability test. During the test, the amplitude of the current peaks was maintained at 9.2–9.5 μA. (Figure 5a) Signs of abrasion on the PFA surfaces were not observed because of its abrasion resistance (Figure 5c,d). We also succeeded in charging a capacitor of 330 μF capacitance up to 3 V in 6 minutes at a speed of 200 mm/s, a damping force of 0.6 kgf, and a displacement of 50 mm to charge the capacitor. (Figure 5b). These results mean that the stable and high output characteristics of STEG, not only do not reduce the reliability of existing vehicle suspensions that require a long life, but also show the possibility of efficiently harvesting mechanical energy, which is wasted by the vibration of a vehicle.

## 4. Conclusions

Through an optimization process and evaluations, we could observe the feasibility of STEG to be applied to shock absorber in the suspension systems of vehicles and become an energy source for low-power sensors for ADAS technology according to their stable and high-output performance. Decisively, a novel, suspension-type, free-standing mode structure and the selection of materials considering both industrial and experimental issues, support the impressive reliability of STEG. Proposing the frictional force as a meaningful parameter related to output performance through an experimental approach, we could determine how frictional force could be adjusted, considering not only output performance, but also the stability of the device. Meanwhile, STEG, which was used in this work, was designed to have 1:8 scaled size compared with the actual size of suspension in a commercial vehicle. Additionally, a vehicle applies tons of damping force to the suspension in practical conditions. Considering the much larger scale and damping force applied to the suspension systems in vehicles, it is expected that a much larger output power can be achieved than the experimental results obtained in the present work.

## Supplementary Materials

The following are available online at http://www.mdpi.com/2072-666X/10/1/17/s1, Figure S1: detailed structure and optical images of STEG, Figure S2: output performance and COMSOL simulation results versus electrode width, Figure S3: Advantages of cylindrical over planar structure with same charge density and area, Figure S4: output performance versus frictional force.
