# Supplementary material for: Cylindrical Free-Standing Mode Triboelectric Generator for Suspension System in Vehicle
Source: Micromachines (Basel). 2018 Dec 29;10(1):17. doi: 10.3390/mi10010017 (PMC6356977; doi:10.3390/mi10010017)
Supplement: Supplementary file 1 [file micromachines-10-00017-s001.pdf]

Supplementary Materials: Cylindrical Free-Standing Mode Triboelectric Generator for Suspension System in Vehicle

Minki Kang, Tae Yun Kim, Wanchul Seung, Jae-Hee Han and Sang-Woo Kim

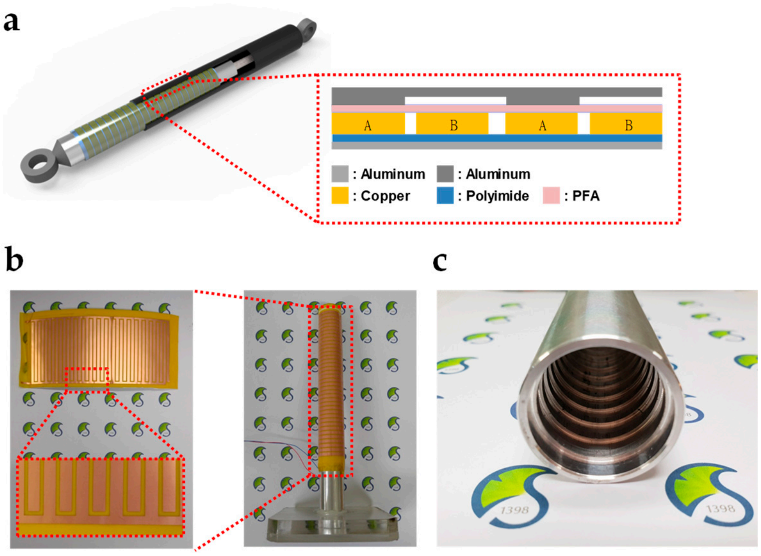

Figure S1. Detailed structure and optical images of STEG: (**a**) the structure and materials of STEG; (**b**) optical images of PFA film and copper electrode on the inner cylinder; (**c**) optical image of the outer cylinder and internal structure.

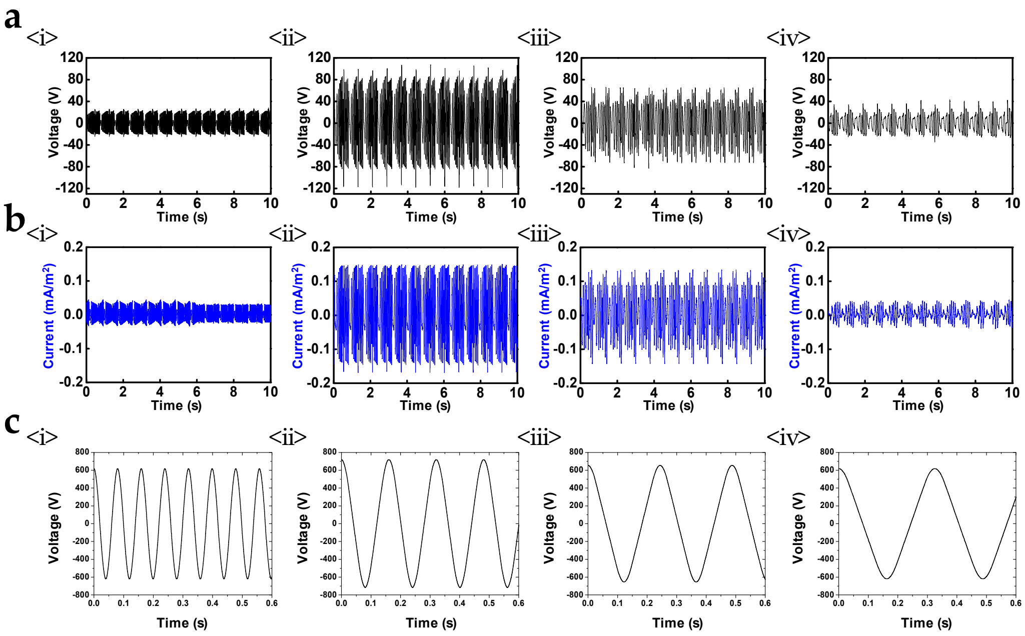

Figure S2. Output performance and COMSOL simulation results versus electrode width: (**a**) output voltage of STEG; (**b**) short-circuit current of STEG; (**c**) COMSOL simulation results for open circuit voltage, resistance was set to 100 MΩ. ((i), (ii), (iii), (iv): results according to electrode width 1, 3, 5, and 7 mm, respectively).

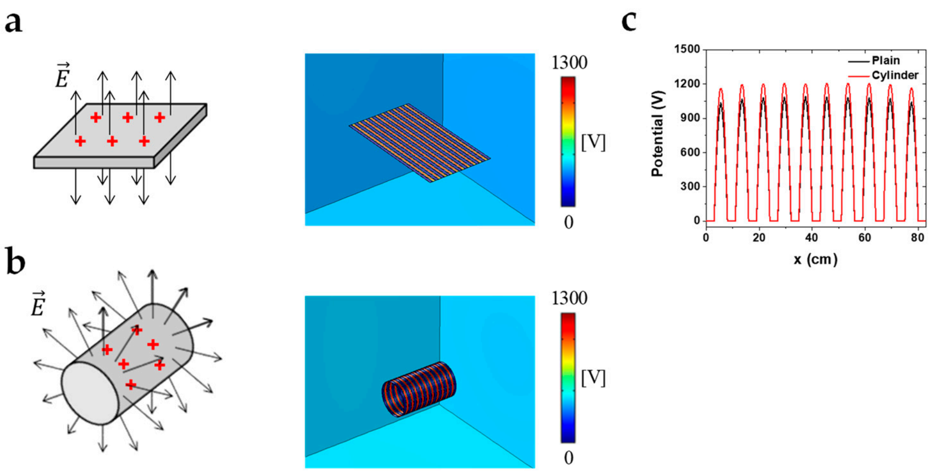

Figure S3. Advantages of cylindrical over planar structure with same charge density and area: (**a**) electric field and potential on planar surface; (**b**) electric field and potential on cylinder surface; (**c**) comparison of open-circuit voltage for each planar and cylindrical model.

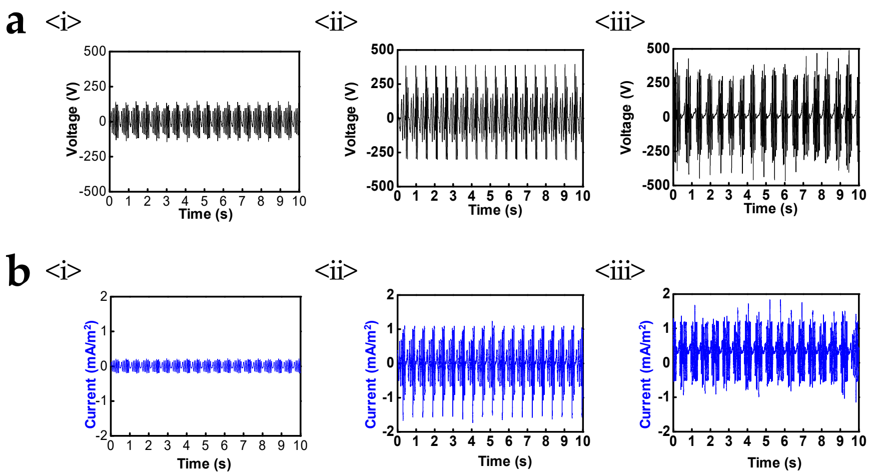

Figure S4. Output performance versus frictional force: (**a**) output voltage of STEG; (**b**) short-circuit current of STEG. ((i), (ii), and (iii): results according to 0.5, 0.6, and 0.9 kgf).
